# Monocyte Caspase-1 Is Released in a Stable, Active High Molecular Weight Complex Distinct from the Unstable Cell Lysate-Activated Caspase-1

**DOI:** 10.1371/journal.pone.0142203

**Published:** 2015-11-24

**Authors:** Obada R. Shamaa, Srabani Mitra, Mikhail A. Gavrilin, Mark D. Wewers

**Affiliations:** Davis Heart and Lung Research Institute, Division of Pulmonary, Allergy, Critical Care and Sleep Medicine, Department of Internal Medicine, Wexner Medical Center, The Ohio State University, 473 W. 12^th^ Avenue, 201 DHLRI, Columbus, OH, United States of America; Virginia Tech University, UNITED STATES

## Abstract

Mononuclear phagocytes utilize caspase-1 activation as a means to respond to danger signals. Although caspase-1 was discovered using highly concentrated cell extracts that spontaneously activate caspase-1, it is now clear that in live cell models caspase-1 activation occurs in the process of its cellular release and is not an intracellular event. Therefore, we compared the characteristics of caspase-1 activation in the cell lysate model to that of caspase-1 that is released in response to exogenous inflammasome activation. Whereas both models generated active caspase-1, the cell-lysate induced caspase-1 required highly concentrated cell lysates and had a short half-life (~15 min) whereas, the activation induced released caspase-1 required 2–3 log fold fewer cells and was stable for greater than 12 h. Both forms were able to cleave proIL-1beta but unexpectedly, the released activity was unable to be immunodepleted by caspase-1 antibodies. Size exclusion chromatography identified two antigenic forms of p20 caspase-1 in the activation induced released caspase-1: one at the predicted size of tetrameric, p20/p10 caspase-1 and the other at >200 kDa. However, only the high molecular weight form had stable functional activity. These results suggest that released caspase-1 exists in a unique complex that is functionally stable and protected from immunodepletion whereas cell-extract generated active caspase-1 is rapidly inhibited in the cytosolic milieu.

## Introduction

Caspase-1 is a protease required for the cleavage of the pro-inflammatory cytokines interleukin-1β (IL-1β) and IL-18 into their active forms [1]. Initially known as interleukin-1β converting enzyme (ICE), caspase-1 was discovered by incubating recombinant proIL-1β with concentrated monocytic lysates to identify the protease responsible for generating mature IL-1β [2, 3]. This biochemical approach, termed the cell-extract, found that caspase-1 is expressed constitutively in myeloid cells and exists in a zymogenic form that spontaneously becomes activated under hypotonic buffer conditions [4, 5]. This cell-extract technique further led to discoveries such as mature caspase-1 enzyme existing as a tetrameric protein that is generated via dimerization of the pro-caspase-1 zymogen facilitated by a 700 kDa complex, termed the inflammasome [6–8].

Mature caspase-1 cleaves its cytokine substrates, IL-1β and IL-18, which disseminate and coordinate components of the innate inflammatory response, such as fever, neutrophil recruitment, endothelial activation, and cytotoxic NK cell activation [9]. In addition to its role in activating cytokines, caspase-1 is involved in an inflammatory type of programmed cell death termed pyroptosis [10]. This form of cell death is marked by an intracellular aggregation of the inflammasome adaptor protein, apoptosis-associated speck-like complex containing a CARD (ASC), termed an ASC speck [11–13]. Morphologic hallmarks of this form of cell death are large plasma membrane blebbing, loss of membrane integrity resulting in a swollen cell, and nuclear fragmentation [10].

The activation of caspase-1 requires the formation of the inflammasome, an intracellular multi-protein complex that assembles upon detection of pathogen or danger associated molecular patterns (PAMP/DAMP) by intracellular pattern recognition receptors (PRR), such as a member of the NOD-like receptor (NLR) family, NLRP1, NLRP3, NLRC4, or other sensors such as AIM2, IFI16, and pyrin [14–16]. Zymogenic caspase-1 is recruited to the inflammasome complex via its N-terminal caspase-associated recruitment domain (CARD) [5, 17]. Recruitment to the inflammasome complex facilitates the formation of caspase-1 homodimers. Following the cleavage of the CARD pro-domain, as well as a small spacer sequence between the 20 kDa (p20) and 10 kDa (p10) subunits, a tetrameric enzyme, (p20/p10)_2_, forms with two active sites on opposing ends of the enzyme [2, 6, 7]. This mature tetrametic form of caspase-1 has the highest affinity for substrates [4, 8].

Of note, it is difficult to detect the mature form of caspase-1 endogenously in the cytosol of monocytes in response to inflammasome agonists [2, 17, 18]. Using immuno-electron microscopy it has been shown that mature subunits of caspase-1 can be detected on the monocytic plasma membrane, with only the zymogenic enzyme detected in the cytosol [19]. Classically, the detection of mature caspase-1 subunits extracellularly serves as a marker for inflammasome activation *in vitro* [20]. This is in contrast to the apoptotic caspases and the other inflammatory caspases (4, 5, and murine ortholog 11) where their mature form and associated activity are detected in the cytosol [21, 22].

Based on the absence of mature caspase-1 activity in the cytosol and the unique fact that mature caspase-1 is released into the supernatant, we elected to study the function of the released caspase-1 compared to the biochemically activated caspase-1 generated by the cell-extract model. We hypothesized that monocytes release mature caspase-1 to function as an extracellular inflammatory protease. We found that released caspase-1 is functionally stable in contrast to the rapid loss of caspase-1 function in the cell-free extract. However, unexpectedly, the functional form of extracellular caspase-1 is detected in a high molecular weight complex. These findings suggest that released caspase-1 may disseminate inflammatory processes beyond the local monocyte milieu.

## Materials and Methods

### Reagents, treatment agents and antibodies

#### Culture media

RPMI-1640 (Corning and Media tech), BEGM (Bronchial Epithelium Growth Medium, Lonza), fetal bovine serum (Atlas), pen-strep (Life technologies). *Reagents*: phosphate buffered saline 0.9% (PBS, Gibco Life Technologies), DTT (dithiothreitol), complete protease inhibitor cocktail (Sigma, P8340), phenylmethylsulfonyl fluoride (PMSF), N-(methoxysuccinyl)-Ala-Ala-Pro-Val-chloromethyl ketone (m-AAPV-cmk), HEPES, Tris base (Fischer Scientific), NaCl, NaF, KCl, MgCl_2_, EDTA, EGTA, Triton X-100, Na-deoxycholate.

#### Agonists, substrates, and inhibitors

Endotoxin (LPS, from *E*. *coli* strain 0111:B4; Alexis Biochemicals, San Diego), ATP, Ac-Trp-Glu-His-Asp-7-Amino-4-trifluoromethylcoumarin (afc) (WEHD-afc, Calbiochem), Ac-Tyr-Val-Ala-Asp-chloromethylketone (cmk) (YVAD-cmk, Calbiochem), Biotin-YVAD-cmk (Anaspec, USA), recombinant human IL-1β (His-tag, Sino Biological, China).

#### Antibodies

Anti-human caspase-1 (R&D mouse monoclonal IgG2A), mouse IgG2A (kappa) isotype control, anti-caspase-1 (lab-generated rabbit polyclonal caspase-1 antiserum (YK)), YK prebleed rabbit serum, anti-ASC (MBL mouse monoclonal IgG1), anti-ASC (lab-generated rabbit polyclonal ASC antiserum (ASCAR)), anti-human IL-18 (MBL International, rabbit monoclonal, IgG2A), anti- human IL-18 (MBL International, mouse monoclonal, IgG2A), biotin-labeled anti-human IL-18 (MBL International, rat monoclonal, IgG2A), recombinant human IL-18 (MBL International), and anti-LAMP1 (Cell Signaling, D2D11, rabbit IgG). Secondary antibodies were: donkey anti-rabbit-HRP & sheep anti-mouse-HRP (ECL, GE Healthcare, UK).

The source of all agents not specified was from Sigma-Aldrich, US.

### Cell lines, culture, and treatments

Whole blood for study was obtained from healthy human donors after written informed consent using a protocol approved by the Ohio State University Institutional Review Board. In selected cases, peripheral blood mononuclear cells were also isolated by Histopaque density gradients from fresh buffy coats provided anonymously by the American Red Cross in an IRB exempt protocol. Monocytes were isolated from peripheral blood mononuclear cells by CD14 positive selection (MACS, Miltenyi Biotec, Auburn,CA). This method of purification yields greater than 98% pure monocytes based on flow cytometry analysis. Monocytes (20x10^6^/ml) were incubated in culture tubes in RPMI-1640 medium supplemented with 10% FBS and 1% pen-strep (RPMI 10/1).

THP1 cells (human acute monocytic leukemia cell line) were from ATCC (lot 385653), and were cultured in RPMI 10/1. THP1 cells were used at mid-log growth phase as determined by cell-culture concentrations between 0.5–1.0x10^6^ cells/ml. Cells were regularly checked for the absence of mycoplasma contamination [23].

THP-1 cells stably expressing YFP-ASC were generated in pLenti plasmid (Invitrogen), as we describe earlier [24]. To knock down endogenous ASC, siASC (5’-AAGCTGACCCT -GAAGTTCA-3’) and siEGFP (as a control) (5’-GGCCTGCACTTTATAGACC-3') were generated with lentiviral constructs, as we described earlier [25]. Resulting plasmids pGreenPuro-siASC and pLenti-siEGFP-RFP were verified by sequencing and then were co-transfected with helper plasmids pMD.G and pCMVΔR8.2 to packaging cell line HEK293FT (Invitrogen) to generate lentivirus. THP-1 cells were transduced with lentivirus and siRNA positive cells, expressing either green or red fluorescence, were sorted two times by flow cytometry (FACSAria, Beckton Dickinson) as we described earlier [16, 26]. Levels of ASC RNA and protein expression were reduced by 90%, as we verified by qPCR and immunoblot.

To over-express proIL-1β, HEK293 were transfected with proIL-1β-EGFP plasmid using PolyJet (SignaGen laboratories) resulting in > 95% alive and bright positive cell yield.

For extracellular caspase-1 analysis, THP1 cells at concentrations of 20x10^6^ cell/ml were used to produce detectable caspase-1 WEHD-afc activity in the supernatant and for Western blot. Cells were cultured in polypropylene tubes (Denville Scientific Inc.) during the experiments and treated with LPS (1μg/ml) to prime cells for 30 minutes and then ATP (5 mM) was added for 30 minutes to activate the inflammasome. To separate cells from the extracellular fraction, samples were spun down at 400g for 5 min. Supernatants were collected and clarified by spinning at 16,000g for 5 min at 4°C. To analyze intracellular caspase-1 in these samples, the cell pellet was washed once with ice cold PBS and spun down at 400g for 5 min. The cells were resuspended in 1ml RPMI-1640 10/1 media supplemented with complete protease inhibitor cocktail (Sigma, 1:100), 1mM PMSF, and 100 μM AAPV-cmk, and lysed by freeze/thaw in liquid nitrogen and a 37°C water bath 3 times. Cells lysates were spun down at 16,000g for 5 minutes at 4°C to remove cellular debris. Lysates and supernatants were then used for caspase-1 activity assays and detection of caspase-1 by Western blot.

To assess extracellular cleavage of pro-IL-1β by caspase-1, supernatants were collected from THP1 cells (2x10^9^cells/ml) stimulated with LPS (1μg/ml) for 30 min and ATP (5 mM) for another 30 min. HEK293 cells expressing pro-IL-1B-EGFP were lysed in CHAPS buffer containing 10mM DTT. From this lysate, 10μg of total protein was added to 18 μl of THP1 supernatants and 14 μl of CHAPS containing 10mM DTT for a total volume of 40 μl and incubated overnight at 37°C in the presence of either YVAD-cmk (100 μM) or caspase-1 negative control, z-FA- fmk (100 μM). The samples were then run on Western blot for detection of proIL-1β cleavage.

### Caspase-1 cell-free extract

THP1 cells were lysed at a concentrations of 1, 2, & 3x10^7^ cells/100 μl in a hypotonic buffer (Buffer W: 20 mM HEPES, 10 mM KCl, 1.5 mM MgCl_2_, 1 mM EDTA, 1 mM EGTA, pH = 7.4, KOH). Briefly, after washing the cells in 1 ml cold PBS, the cell pellet was resuspended in 100 μl of cold Buffer W supplemented with complete protease inhibitor cocktail (Sigma, 1:100), 1 mM PMSF, and 100 μM AAPV-cmk, and allowed to swell on ice for 10 minutes under these hypotonic conditions. Lysis was accomplished by 10 slow- strokes using a 28½g needle on a tuberculin syringe while on ice. Cells were spun at 16,000g for 15 minutes at 4°C in a bench top centrifuge to remove nuclei and large subcellular structures. Supernatants were placed into pre-chilled Eppendorf tubes and kept at 4°C prior to caspase-1 cleavage and activity assays. Cell-extract samples were either directly loaded from 4°C or pre-incubated at 37°C for various periods of time and all samples were loaded simultaneously into the fluorimeter. Caspase-1 activity was assessed by taking the maximal slope of fluorescence obtained within 1h.

### Caspase-1 activity assay

Cleavage of the caspase-1 fluorogenic tetrapeptide substrate (WEHD-afc) was used to assess functional caspase-1 activity. Briefly, 50 μl of sample was added to 50 μl of assay buffer (Buffer W + 100 μM WEHD-afc + 10 mM DTT) in a 96-well black Costar plate (Fischer Scientific) and activity was measured using a spectrophotometer CytoFluor^®^ Series 4000 Fluorescence Multi-Well Plate Reader (excitation 360/40 nm, emission 460/40 nm). Readings were taken every 30 seconds or every minute for 60–120 min or for 12h and slopes were calculated over the linear portion of the curves. AFU = arbitrary fluorescent units are presented.

### ASC speck visualization using YFP-ASC THP1 cells

For microscopy, 10^6^ cells/ml THP1 cells stably expressing YFP-ASC were plated in 6-well culture plates (Costar, flat bottom polystyrene) in a 2ml volume. Cells were treated with respective agonists and ASC specks were visualized using an Olympus IX50 inverted microscope using 480/20 nm excitation and detection at 520 nm emission under a 20X objective. ASC specks were quantified from the fluorescent images using Image J software (NIH, http://rsbweb.nih.gov/ij).

### Lactate dehydrogenase (LDH) cytotoxicity assay

LDH release from the cell was used as an indicator of cell death using an NAD^+^ reduction assay (Roche Applied Science). Supernatants from treated cells were collected, clarified by centrifugation at 400g for 5 min, and used for LDH assay. For a positive control, total LDH content in untreated THP1 cells was obtained by lysing cells with 1% Triton X-100. RPMI-1640 media was used as a blank and OD values were subtracted from readings of samples and positive control. LDH concentration in the medium was measured at 490 nm. Cell death was calculated by the formula: cytotoxicity (%) = [(sample-blank)/(positive control-blank) x 100].

### Interleukin-18 ELISA

Released IL-18 was quantified by sandwich ELISA using MBL antibodies. Anti-human IL-18 (MBL International, mouse IgG2A monoclonal) was coated on 96-well clear Costar plate (Fischer Scientific) at 1:1000 overnight at 4°C. Plates were blocked with 5% bovine serum albumin, followed by incubation of samples and recombinant IL-18 standard (MBL International). Biotin-labeled anti-human IL-18, 1:1000 (MBL International, rat IgG2A monoclonal) was then added. Each step was incubated for a minimum of 1h with 4 washes using PBS + 0.5% Tween 20. Streptavidin-HRP (eBiosciences) was added for 1h and after washing, the plate was developed using TMB Peroxidase Substrate and Peroxidase Substrate Solution B (KPL, SeraCare Life Sciences). Plates were read on a Perkin Elmer 2030 Victor X3 Multilabel Reader, measuring absorbance at 450 nm after subtracting background 630 nm absorbance.

### Biotin-YVAD-cmk labeling of caspase-1

Biotinylated-YVAD-cmk was used to label the active site of caspase-1. Samples from each treatment were labeled with biotin-YVAD-cmk (50 μM) by incubating for 1h at 37°C. Samples were then boiled in Laemmli buffer and analyzed by Western blot with 10% BSA used to block the PVDF membrane. Detection of biotinylated proteins was performed using a Vectastain Elite ABC kit (Vector Labs, US)–Reagents A (avidin DH) and Reagent B (biotinylated-horseradish peroxidase) according to manufacturer’s instructions. Briefly, 2 drops of Vectastain reagent A and 2 drops of reagent B were mixed in 10 ml TBS-1% Tween-20 and allowed to incubate for 30 minutes. This AB reagent (1X stock) was diluted to 0.01X with TBS-1% Tween-20 and incubated with PVDF membrane for 30 min under agitation. Enhanced chemiluminescence solution (Amersham, GE Life Sciences) was used to detect biotinylated bands.

### Immunoprecipitation (IP) of ASC and caspase-1

Extracellular ASC and caspase-1 was immunoprecipitated from the clarified supernatants by dividing samples and incubating each treatment with either anti-ASC (1:62.5, MBL mouse monoclonal) with control mouse IgG1 (Selenus) or anti-human ICE (1:250, caspase-1, R&D mouse monoclonal) with control mouse IgG2A (kappa, Sigma), respectively, in an orbital rocker at 4°C for 2h. Protein A/G beads (50 μl, Thermo Fisher Scientific) were washed with 1 mL of cold PBS and added to the samples and incubated for another 2h in an orbital rocker at 4°C. Samples were spun down at 16,000g for 5 min at 4°C to pellet immunoprecipitate-bound beads. Non-immunoprecipitated supernatants were collected for an additional round of immunoprecipitation, as well as for WEHD-afc activity assay and Western blot. Immunoprecipitate beads were washed 3 times in 1mL cold PBS by spinning down at 16,000g for 5 min at 4°C. For Western blotting, beads or supernatants were boiled in Laemmli buffer. Second round of immunoprecipitation was either performed the same day or allowed to incubate with the antibody overnight at 4°C in an orbital rocker with the beads added the next day. Co-immunoprecipitation of caspase-1 with ASC was probed on immunoblot with YK antibody.

### Isolation of microvesicles from samples

Differential centrifugation was used to isolate microvesicles from the extracellular fraction. Supernatants from LPS/ATP treated THP1 cells were clarified by spinning at 400g and then at 16,000g for 5 min. The resulting supernatant was spun at 100,000g for 60 minutes to isolatethe microvesicle pellet according to [27].

### Size exclusion chromatography for analysis of released caspase-1

To assess the size of released caspase-1, 20 ml of media from LPS/ATP treated THP1 cells (2x10^7^ cells/ml) was pooled and concentrated using Amicon Ultra 4 mL and 2mL Centrifugal Filters (3kDa and 10 kDa cut-offs) (Millipore). Concentrators were spunin a bench-top swinging bucket centrifuge at 3,500g for 4h at 4°C. Concentrated supernatants were resuspended in column buffer (20mM HEPES, 10% glycerol, pH = 7.5, NaOH) and 2 ml of this concentrated media was loaded onto a HiPrep 16/60 Sephacryl S-200 HR (GE Healthcare Life Sciences) gel chromatography column using a **GE Pharmacia AKTA FPLC Purifier 10** (GE Healthcare Life Sciences) at a flow rate of 0.5ml/min. Column fractions were collected in 2 ml volumes for analysis. Gel chromatography was performed by the Campus Chemical Instrument Center (CCIC) Mass Spectrometry and Proteomics Facility at the Ohio State University.

### Caspase-1 ELISA

Detection of caspase-1 in column fractions was performed using Quantikine^®^ Human Caspase-1/ICE ELISA kit (R&D systems) according to manufacturer’s instructions. Plates were read on a Perkin Elmer 2030 Victor X3 Multilabel Reader, measuring absorbance at 450 nm after subtracting background 630 nm absorbance.

### Western-blot detection of proteins

For the *in vitro* caspase-1 analysis, 50 μl of lysate or supernatant was loaded onto 10% Bis-Tris SDS gel (NuPAGE Novex, Life Technologies) after denaturing the proteins in Laemmli buffer. Due to the release of β-actin and ASC, blots are normalized to total volume (50 μl or 5% of total sample). For cell-extract lysates, 15–20μg of total protein was boiled in Laemmli buffer and loaded onto a 10% Bis-Tris SDS gel. Gels were run in MOPS SDS buffer (NuPAGE MOPS SDS Running Buffer (20X), Life Technologies). Magic Mark^™^ XP Western Standard (Invitrogen) was used as a molecular weight marker. Proteins were transferred onto PVDF membranes in Tris-glycine buffer (20% methanol, Tris-glycine (10X), Bio-Rad). Membranes were blocked in 10% non-fat dry milk (TBS (20X) + 0.1% Tween 20) or 10% bovine serum albumin (TBS = 0.1% Tween 20) for the Biotin-labeling experiments. Anti-caspase-1, anti-IL-18, anti-ASC, anti-LAMP1 (1:1,000) and beta-actin (1:10,000) antibodies were used to probe gels, and respective secondary antibodies (1:10,000) were subsequently added. Membranes were rinsed and washed 3 times with TBS + 0.1% Tween-20 between each of the incubation steps. Enhanced chemiluminescence solution (Amersham, GE Life Sciences) was used to detect labeled proteins and blots were developed using HyBlot CL autoradiography film (Denville Scientific Inc.) on a Konica Minolta SRX-101A film processor (Konica Minolta Medical Imaging U.S.A., Inc.).

### Protein Concentration Estimation

Protein concentration in samples was measured using the *DC* Protein Assay Kit (BioRad) according to manufacturer’s instructions. Samples were read at 750 nm using Beckman DU 640B spectrophotometer.

### Statistics

GraphPad Prism V was used for statistical analysis. Student’s *t*-test was used for comparison between two groups, and one-way ANOVA was used for multiple group comparison with Tukey’s Multiple Comparison post-hoc test to analyze significant differences. p ≤ 0.05 was considered to be significant. All experiments were performed a minimum of 3 independent times (n = 3) and results are expressed as mean values ± SEM unless specifically noted in figure legend.

## Results

### Active caspase-1 generated by concentrated monocytic lysates is rapidly inhibited

**Fig 1:**
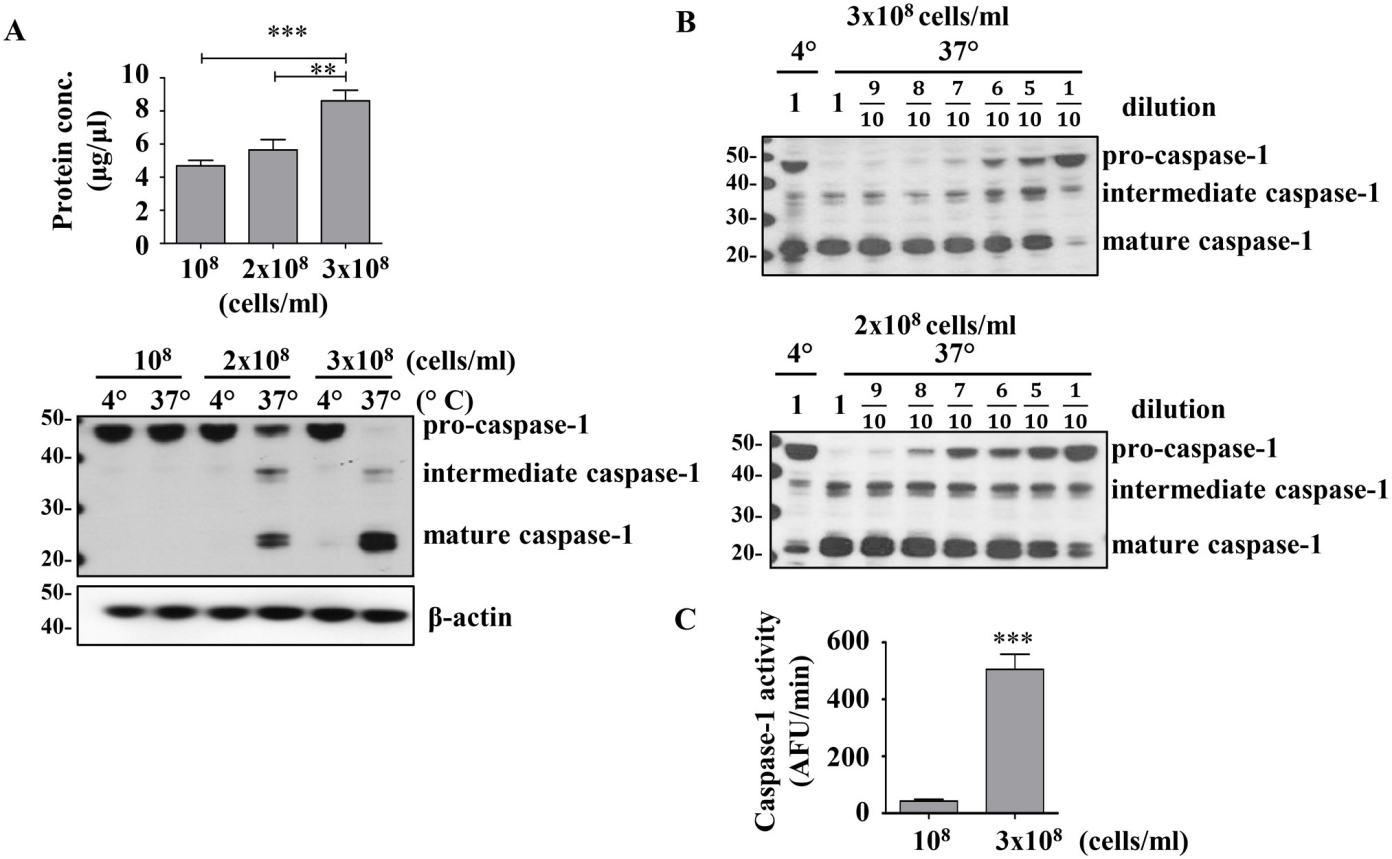
Activation of caspase-1 is dependent on protein concentration. A) Total protein yield (top) and caspase-1 cleavage (bottom) resulteing from 10^8^, 2x10^8^, and 3x10^8^ THP1 cells/ml lysed in hypotonic buffer. To measure caspase-1 activation the cell lysate was incubated at 4°C or 37°C for 1h before immunoblotting. B) Cell-extract from 2x10^8^ and 3x10^8^ THP1 cells/ml were diluted and incubated at 37°C for 1h. Immunoblot shows pro-caspase-1 (45 kDa subunit), intermediate caspase-1 (35 kDa subunit), and mature caspase-1 (20 kDa subunit). C) Caspase-1 activity as measured by WEHD-afc cleavage in 10^8^ and 3x10^8^ THP1 cells/ml. **: *p<0*.*0045*, ***: *p< 0*.*0001 (ANOVA (A)*, *t-test (C)*. Data are expressed as mean ± SEM for n = 8 (A) n = 9 (C) independent experiments and a representative gel from n = 3 (A), n = 1 (B top) and n = 2 (B bottom).

We have previously noted that human monocytes and macrophages release mature IL-1β in a caspase-1 dependent manner without detectable active intracellular caspase-1 [17]. This finding suggested the possibility that functional intracellular caspase-1 levels were either below detection or rapidly inhibited. In order to address this phenomenon we adopted a biochemical approach to generate mature, functional caspase-1. This cell-extract method utilizes concentrated THP1 monocytic cells which are lysed on ice in a hypotonic buffer to generate active caspase-1, detected by the 20 kDa (p20) subunit [4, 8]. Concentrating the lysate to over 8 μg/μl of total protein and incubating at 37°C results in spontaneous production of mature caspase-1(Fig 1A). The concentration-dependence of this model can be reproduced by taking concentrated lysates from two different cell concentrations and diluting them before incubating at 37°C (Fig 1B). The generation of mature caspase-1 by a two-fold increase in total protein concentration, generated from 10^8^ cells/ml vs 3x10^8^ cells/ml, induces a twelve-fold increase in caspase-1 activity as detected by the cleavage of the fluorogenic caspase-1 substrate, WEHD-afc (Fig 1C).

**Fig 2:**
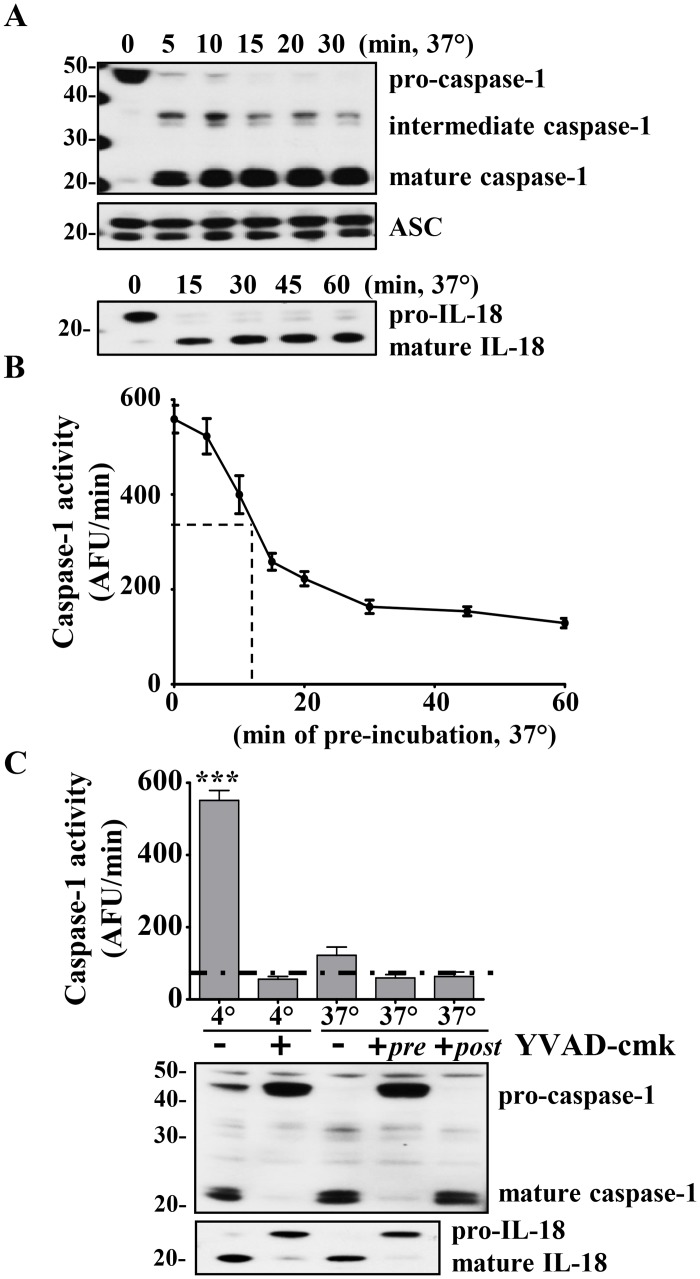
Caspase-1 activity in cell-extract decreases over time. A) Rapid cleavage of caspase-1 and IL-18 into their mature forms during incubation of cell extract from 3x10^8^ THP1 cells/ml at 37°C. ASC was used as a loading control. B) Cell-extracts were pre-incubated at 37°C for various time points and then loaded simultaneously into the fluorimeter and caspase-1 activity (measured as maximal slope: AFU/min) was measured. Dashed line denotes half-life. C) YVAD-cmk (50 μM) inhibits caspase-1 activity and cleavage. Cell-extracts were incubated at 4 and 37°C in presence or absence of YVAD-cmk for 1h and WEHD-afc cleavage measured (AFU/min). Non-YVAD-cmk inhibitable baseline activity is indicated below the dashed-line. Immunoblots for caspase-1 activation and cleavage of endogenous IL-18 was used to confirm caspase-1 specific activity. ***: *p<0*.*0001(ANOVA)*. Representative gels from n = 4 and n = 3 independent experiments (A and C, respectively). Data are expressed as mean ± SEM for n = 4 (B) and n = 3 (C) independent experiments.

This method for activation of caspase-1 occurs rapidly, with complete cleavage of pro-caspase-1 within 15 min (Fig 2A). The specificity of this caspase-1 activity is confirmed by the cleavage of the constitutively expressed substrate proIL-18 to mature IL-18 within 15 min (Fig 2A). In addition, by using this activation method, we found that pre-incubating the cell-extract at 37°C for different periods of time causes the mature caspase-1 to rapidly lose WEHD-afc cleavage activity with a t_1/2_ = 15 minutes (Fig 2B). This finding initially led us to conclude that cytosolic mature caspase-1 is unstable, as also noted elsewhere [28, 29].

Pre-incubation of the cell-extract with the tetrapeptide irreversible caspase-1 inhibitor, YVAD-cmk, blocked maturation of caspase-1, as well as the cleavage of proIL-18 (Fig 2C ***blot***). The YVAD-cmk treated cell-extract samples continued to maintain low level background WEHD-afc activity despite absence of the generation of p20 caspase-1 (Fig 2B, ***graph***). We defined this YVAD non-inhibitable WEHD-afc activity as nonspecific cleavage as has been previously considered [28]. In support of this definition, the background level of non-inhibitable WEHD-afc activity in these fractions is similar to that found in the 10^8^ THP1 cells/ml lysate where no p20 caspase-1 is detected (Fig 1C).

### Rapid release of mature caspase-1 and induction of pyroptosis in response to LPS and ATP treatment

Although it has now been generally accepted that mature caspase-1 is released along with processed IL-1β and IL-18 during inflammasome activation [20, 30], the functional status of this released form of caspase-1 has not been studied. We therefore studied *in vitro* monocyte caspase-1 release in response to the classic inflammasome activators, endotoxin (LPS) and adenosine triphosphate (ATP).

**Fig 3:**
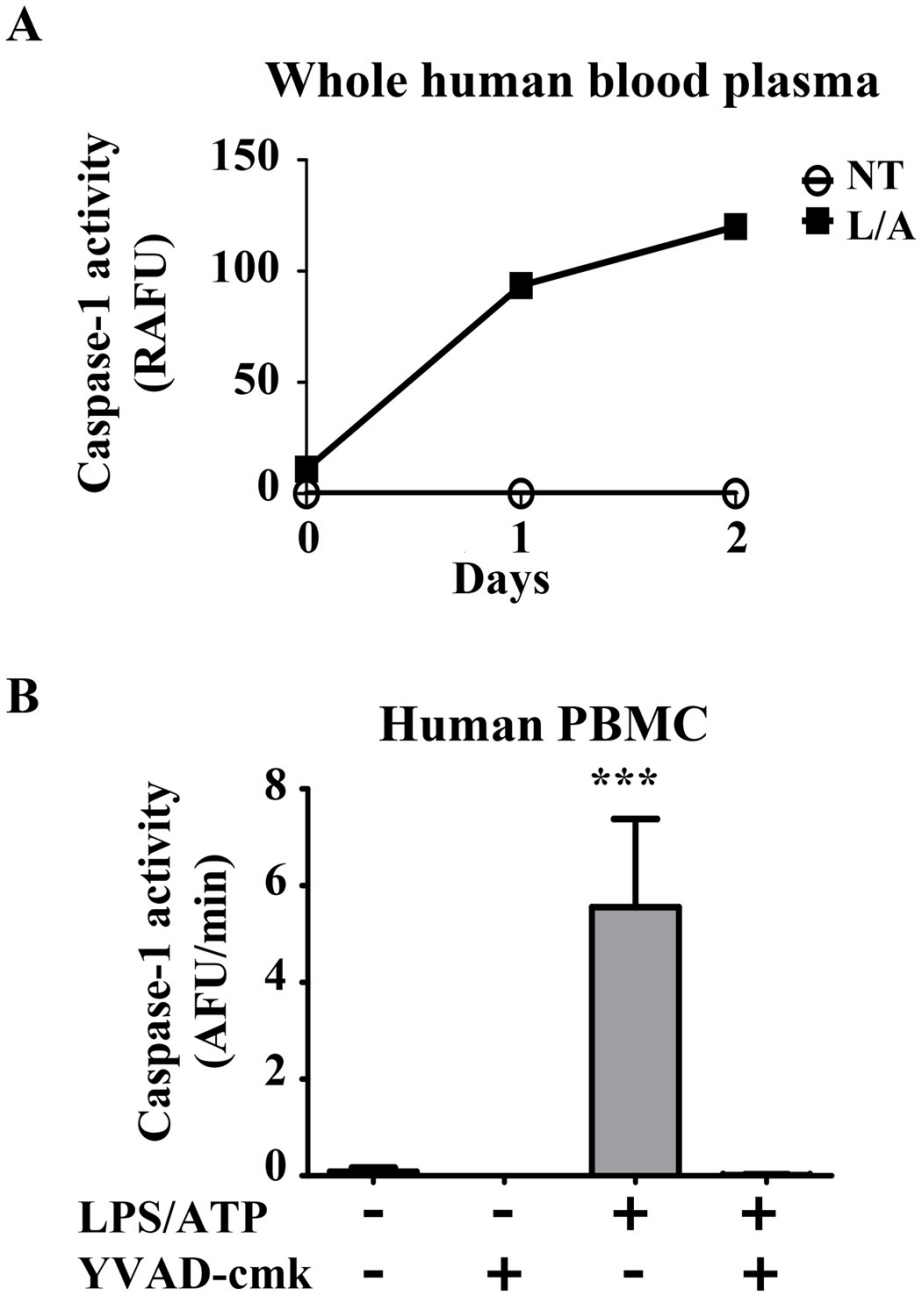
Caspase-1 activity detected extracellularly in whole human blood and purified human monocytes. A) Whole human blood (1 ml) from healthy donors was either left untreated (NT) or stimulated for 30 min with LPS (1 μg/ml) followed by an additional 30 min with ATP (5 mM) (L/A). Plasma was separated from blood cells and incubated with WEHD-afc (50 μM) for 2 days. Activity was calculated by subtracting background (relative absorbance fluorescence unit, RAFU). B) Human monocytes (peripheral blood mononuclear cells (PBMC)) were purified from healthy donors and treated at 2x10^7^ monocytes/ml with LPS (1 μg/ml) and ATP (5 mM) as in *(A)*. Supernatants were isolated and incubated with WEHD-afc (50 μM) in the presence or absence of YVAD-cmk (50 μM) to determine caspase-1 specific activity. Mean slopes over 2h are plotted from n = 2 independent experiments (A). Mean slopes over 2h are plotted with SEM from n = 5 independent experiments (B). ***: *p<0*.*0001(ANOVA)*.

Using whole blood from healthy donors, we treated a 1 ml fraction of whole human blood with LPS for 30 min followed by ATP for an additional 30 min. After separating the plasma from the blood cells, we incubated the plasma with WEHD-afc and detected stable caspase-1 activity for 2 days (Fig 3A). Whole blood only contains 2x10^5^ monocytes/ml, so we next sought to enrich the detection of caspase-1 by isolating and purifying monocytes from healthy donors. Increasing the concentration of human monocytes to 2x10^7^ monocytes/ml generated robust extracellular caspase-1 activity in response to LPS and ATP which was completely inhibited by YVAD-cmk (Fig 3B).

**Fig 4:**
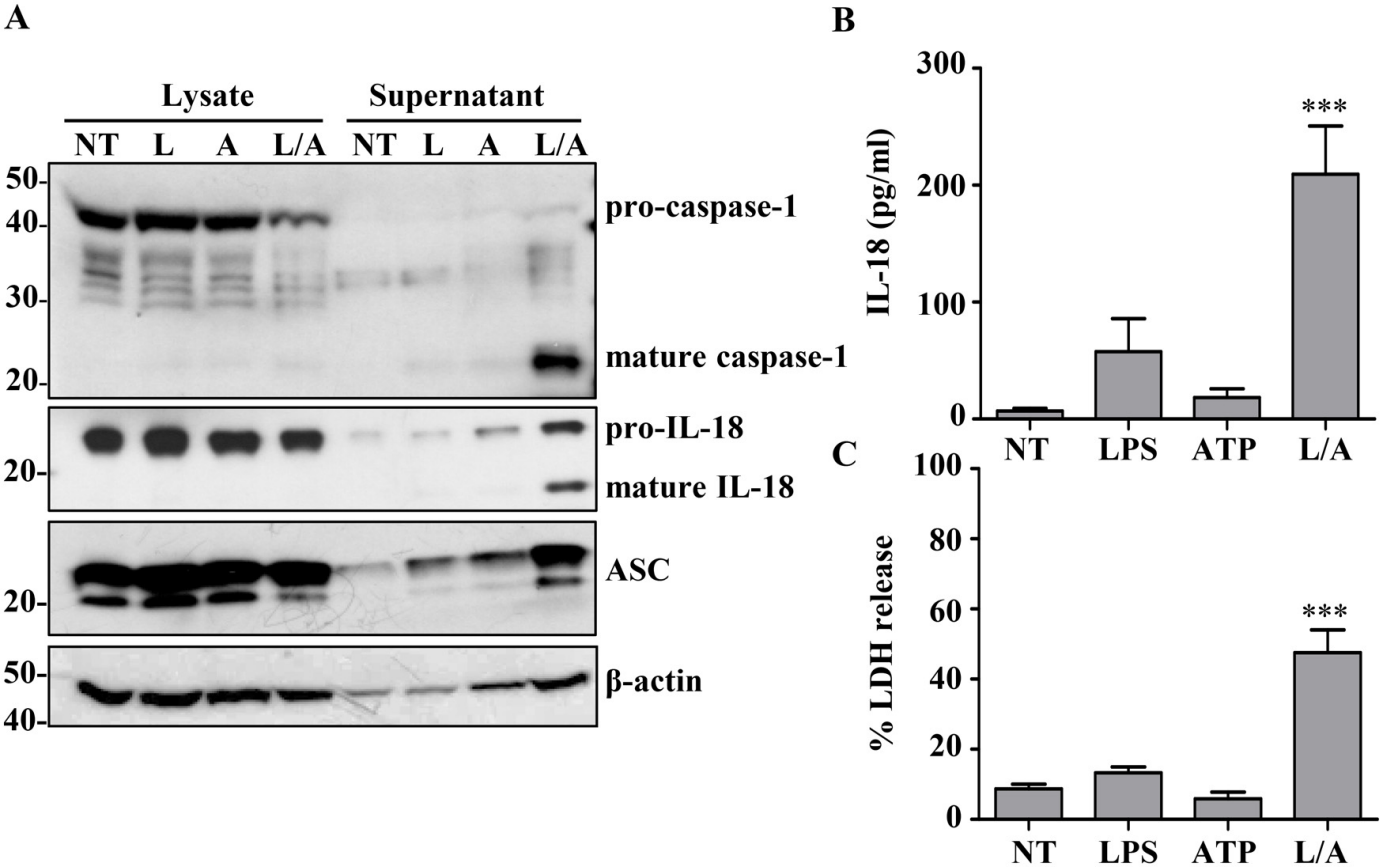
LPS/ATP treatment induces rapid release of inflammasome components and pyroptosis. A) THP1 (2x10^7^ cells/ml) were treated with LPS (1 μg/ml) for 30 min followed by an additional 30 min incubation with ATP (5 mM). Lysates and supernatants were probed for mature forms of caspase-1 and IL-18. (NT = Untreated, L = LPS, A = ATP, L/A = LPS/ATP). β-actin was used as internal control. B) Detection of mature IL-18 release into the supernatant. C) Relative cytotoxicity of treatments by LDH release. ***: *p< 0*.*0001*, *(ANOVA)*. Representative gel from n = 3 independent experiments, and data from n = 10 and n = 6 experiments, respectively.

Treatment of THP1 cells with LPS and ATP also generated robust release of mature caspase-1 (Fig 4A). The concomitant release of processed and preprocessed forms of IL-18 confirmed caspase-1 activation (Fig 4A and 4B). In contrast, intracellular caspase-1 and IL-18 remain in their precursor forms (Fig 4A). The adaptor protein ASC was also released from the cell, in addition to β-actin, under these inflammasome activating conditions (Fig 4A). Assessment of cytotoxicity through the release of lactate dehydrogenase (LDH) [31] supported pyroptosis in these cells (Fig 4C).

**Fig 5:**
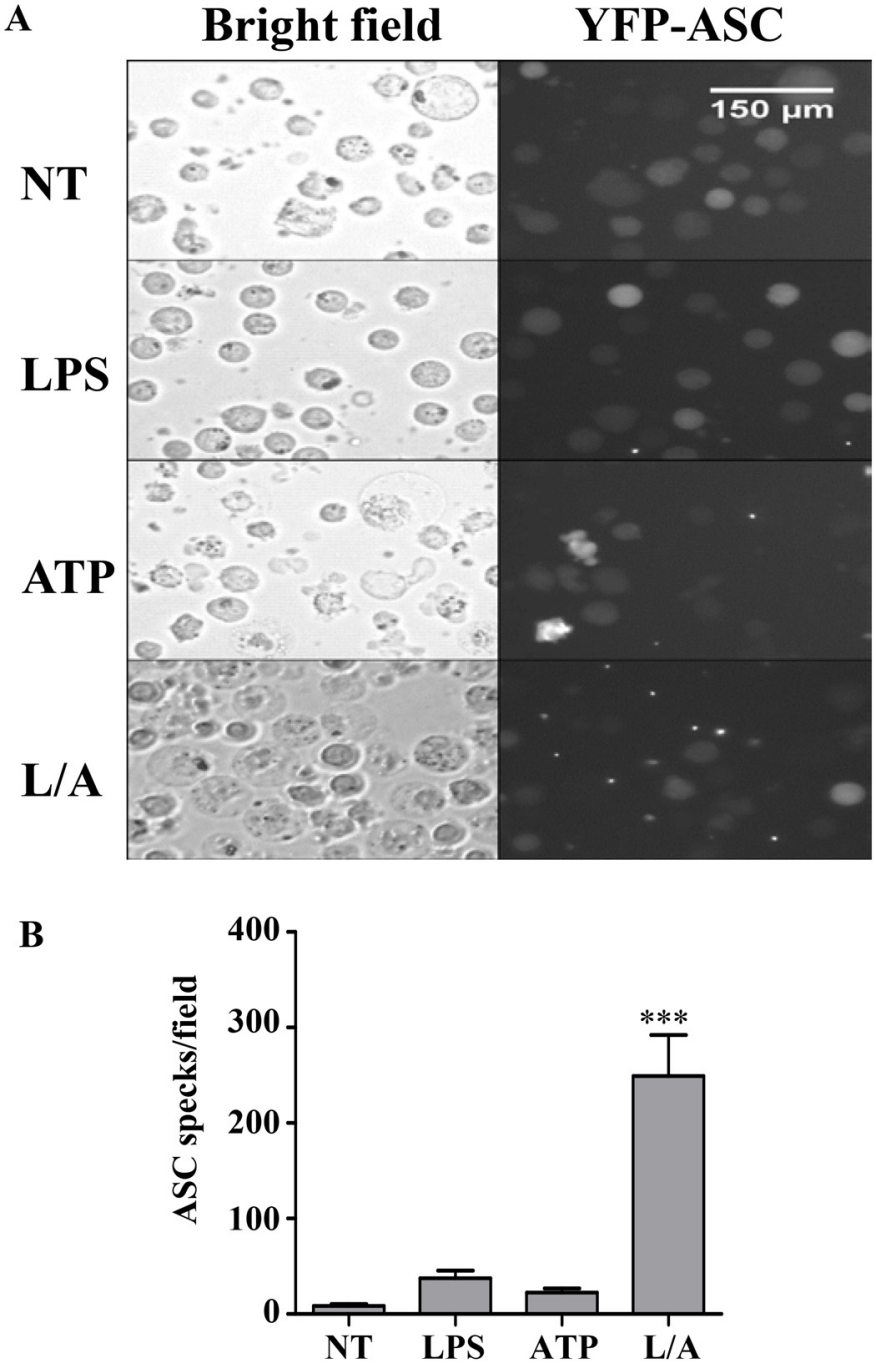
LPS/ATP stimulation induces the intracellular formation of ASC specks as a marker of inflammasome activation. A) YFP-THP1 cells (10^6^ cells/ml) were treated with LPS (1 μg/ml) for 30 min followed by an additional 30 min incubation with ATP (5 mM) to assess the induction of pyroptosis. Bright-field and fluorescent images showing intracellular YFP-ASC specks and loss of membrane integrity are shown. B) Quantification of ASC speck formation in microscopic field under 200X magnification. Representative images and data expressed as mean ± SEM from n = 5 independent experiments.

The induction of pyroptosis by caspase-1 activation was confirmed using THP1 cells that express YFP-tagged ASC allowing the demonstration of intracellular ASC speck formation [11, 12, 25]. We observed that in response to both LPS and ATP, THP1 cells rapidly undergo massive membrane blebbing followed by loss of membrane integrity and cell-swelling that results in the formation of intracellular ASC specks (Fig 5A). In contrast, only low levels of intracellular ASC specks were formed in response to LPS or ATP alone (Fig 5B), despite the high rate of membrane blebbing observed in cells treated with ATP alone (Fig 5A).

### Released mature caspase-1 is functionally stable

**Fig 6:**
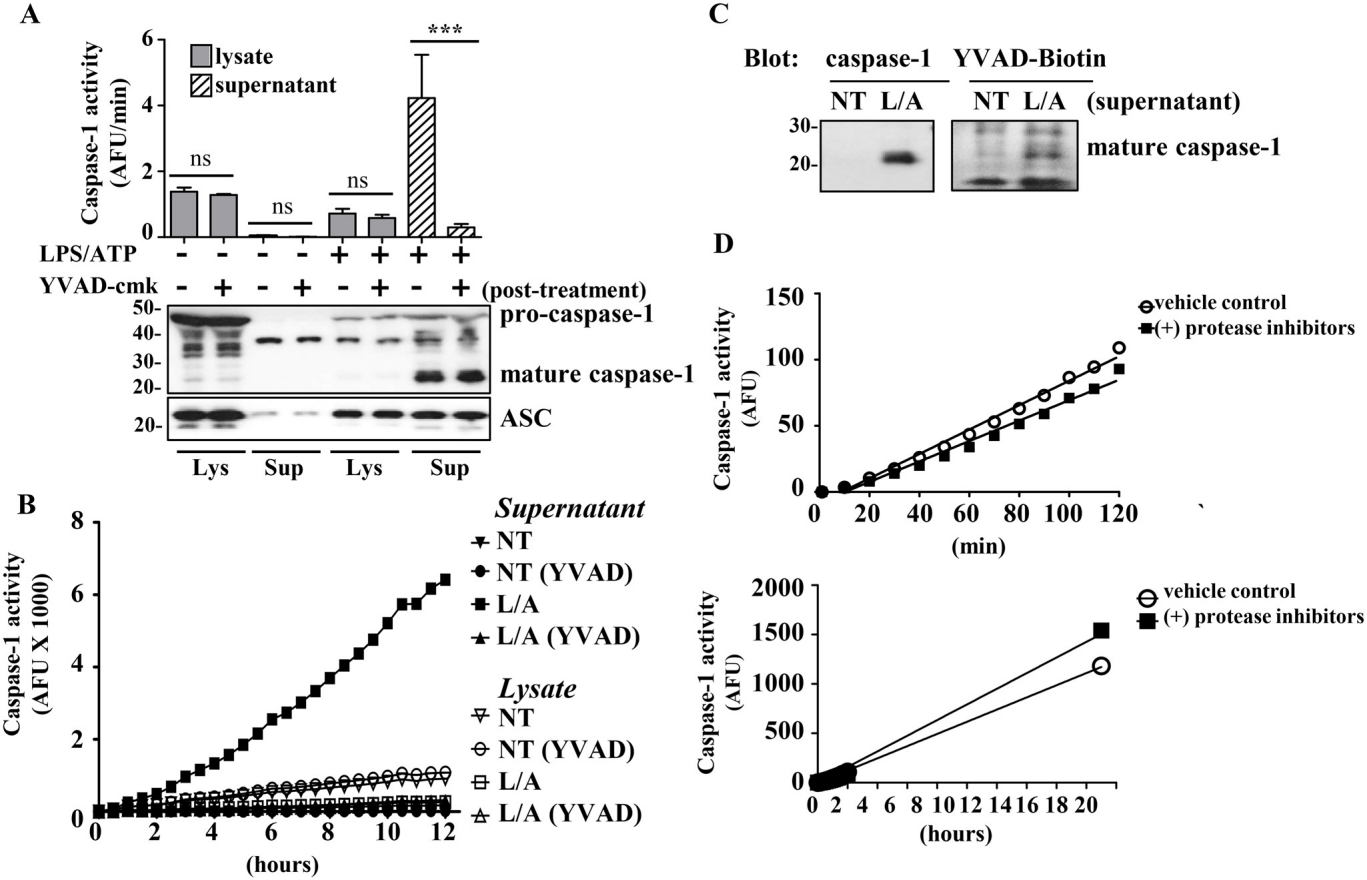
Active caspase 1 is released from THP1 cells upon LPS/ATP stimulation. A) WEHD-afc cleavage in untreated or LPS/ATP treated cell lysates and supernatant (2x10^7^ cells/ml) in the presence or absence of YVAD-cmk inhibitor (50 μM). Caspase-1 presence in samples was verified by Western blotting. ASC used as a loading control *(bottom)*. B) Caspase-1 activity in untreated (NT) and LPS/ATP treated cell lysates and supernatants in the presence or absence of YVAD-cmk inhibitor (50 μM) over the course of 12h. Closed symbols indicate supernatant fractions, open symbols indicate lysate fractions. C) Biotin-YVAD-cmk (50 μM) labeling of mature caspase-1. Supernatants from THP1 cells left untreated (NT) or stimulated with LPS/ATP (L/A) were incubated with biotin-YVAD-cmk. Western blot for caspase-1 is shown on the left, and Vectastain blot for biotinylated proteins on the right. D) Supernatants from LPS/ATP treated THP1 cells supernatant (2x10^7^ cells/ml) were incubated with complete protease inhibitor cocktail or vehicle controls and activity was measured over 2h and overnight. Representative gels and data expressed as mean ± SEM from n = 3 independent experiments *(A*: *ANOVA with Tukey Multiple Comparison test*: ***p<0.*0003)*. Representative time course from n = 3 independent experiments (B) and representative Vectastain blot from n = 3 experiments (C). Mean of n = 2 independent experiments (D).

Because mature p20 caspase-1 is the functional form [2, 4, 5, 8], we sought to confirm that the LPS/ATP released caspase-1 was functional. Indeed the induced supernatant showed caspase-1 activity by WEHD-afc which was completely inhibited by YVAD-cmk (Fig 6A). As expected, the *in vitro* cell lysates which were lysed at 2x10^7^ cells/ml demonstrated no specific caspase-1 activity (Fig 6A). Interestingly, unlike the very short half-life of the cell-extract induced caspase-1 activity (Fig 2), the *in vitro* released supernatant caspase-1 maintained its WEHD-afc activity for over 12h (Fig 6B).

We confirmed that the YVAD-cmk inhibition in the supernatant was specific for caspase-1 by using the biotinylated irreversible caspase-1 inhibitor, biotin-YVAD-cmk, to label the active site of caspase-1. The biotin-labeled p20 band, which contains the caspase-1 active site Cys^285^, suggested that the WEHD-afc activity in the supernatant was due to caspase-1. However, in the context of the non-specific bands bound by the streptavidin/biotin complex the labeling of another 20 kDa protease cannot be excluded.

To further confirm that the loss of caspase-1 activity in the cell-extract was not due to the protease inhibitor cocktail, we treated the extracellular fractions with the protease inhibitor cocktail and assessed caspase-1 function. The protease inhibitors used in the cell-extract did not affect caspase-1 activity, confirming that the loss of function in the cell-extract is intrinsic to the model (Fig 6D).

**Fig 7:**
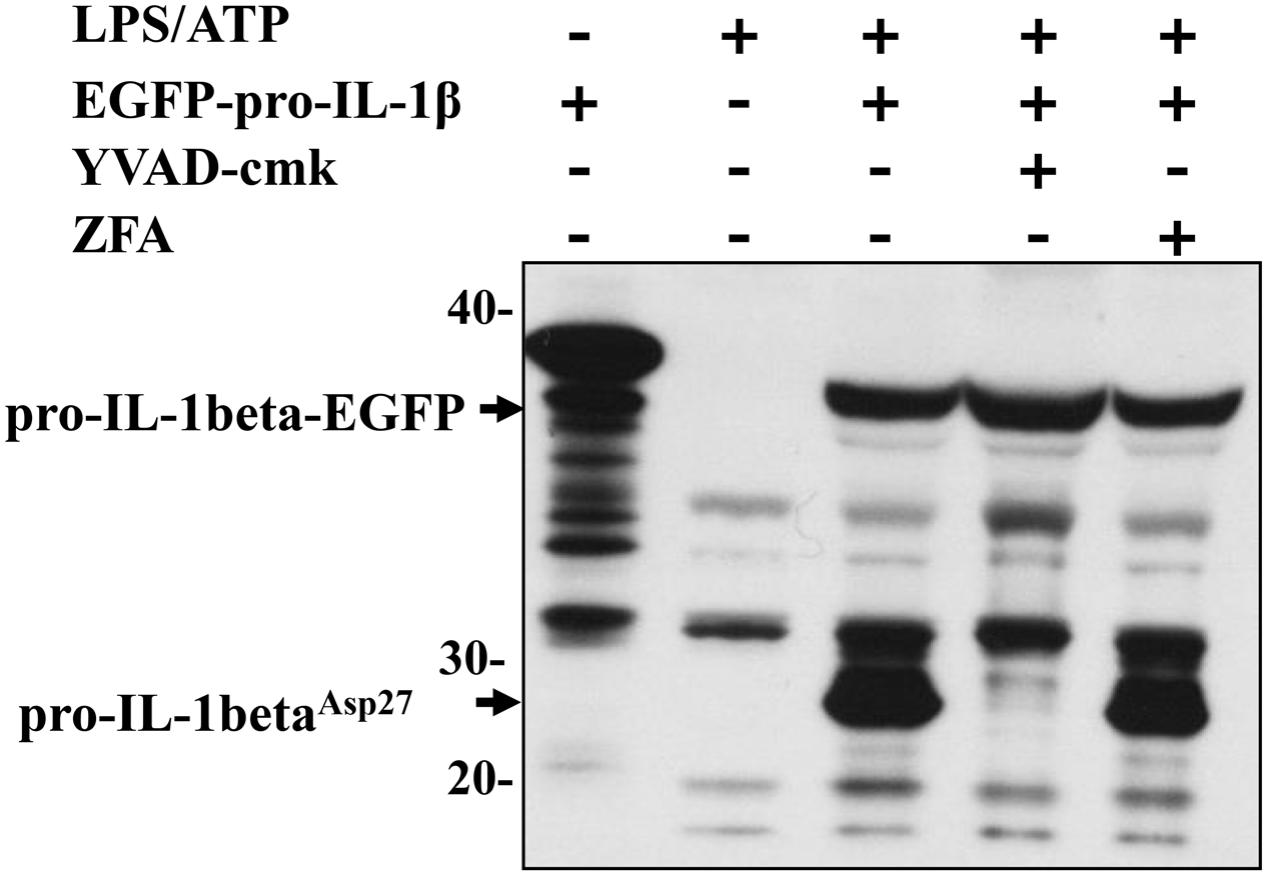
Extracellular caspase-1 is able to cleave pro-IL-1β. THP1 cells (2x10^9^ cells/ml) were left untreated or stimulated with LPS/ATP for 1h and then supernatants were incubated with lysates from HEK293 cells expressing proIL-1β-EGFP for 12h at 37°C in the presence or absence of YVAD-cmk (100 μM) or z-FA-fmk (100 μM apoptotic caspase inhibitor, negative control) and immunoblotted for IL-1β. Representative gel from n = 3 independent experiments. Asp^27^cleavage product of EGFP-proIL-1β detected.

Extracellular caspase-1 activity was confirmed by its ability to cleave its relevant cytokine substrate, we incubated the cell supernatants containing released caspase-1, with recombinant IL-1β from IL-1β-transfected HEK cell lysates. Extracellular caspase-1 was able to generate p28 proIL-1β, the product of the first caspase-1 cleavage step at Asp^27^ (Fig 7) [32]. The extracellular cleavage of recombinant pro-IL-1β by caspase-1 was completely inhibited by YVAD-cmk, confirming that caspase-1 was responsible for the cleavage of proIL-1β.

### Extracellular caspase-1 activity is separate from extracellular ASC and microvesicular caspase-1

**Fig 8:**
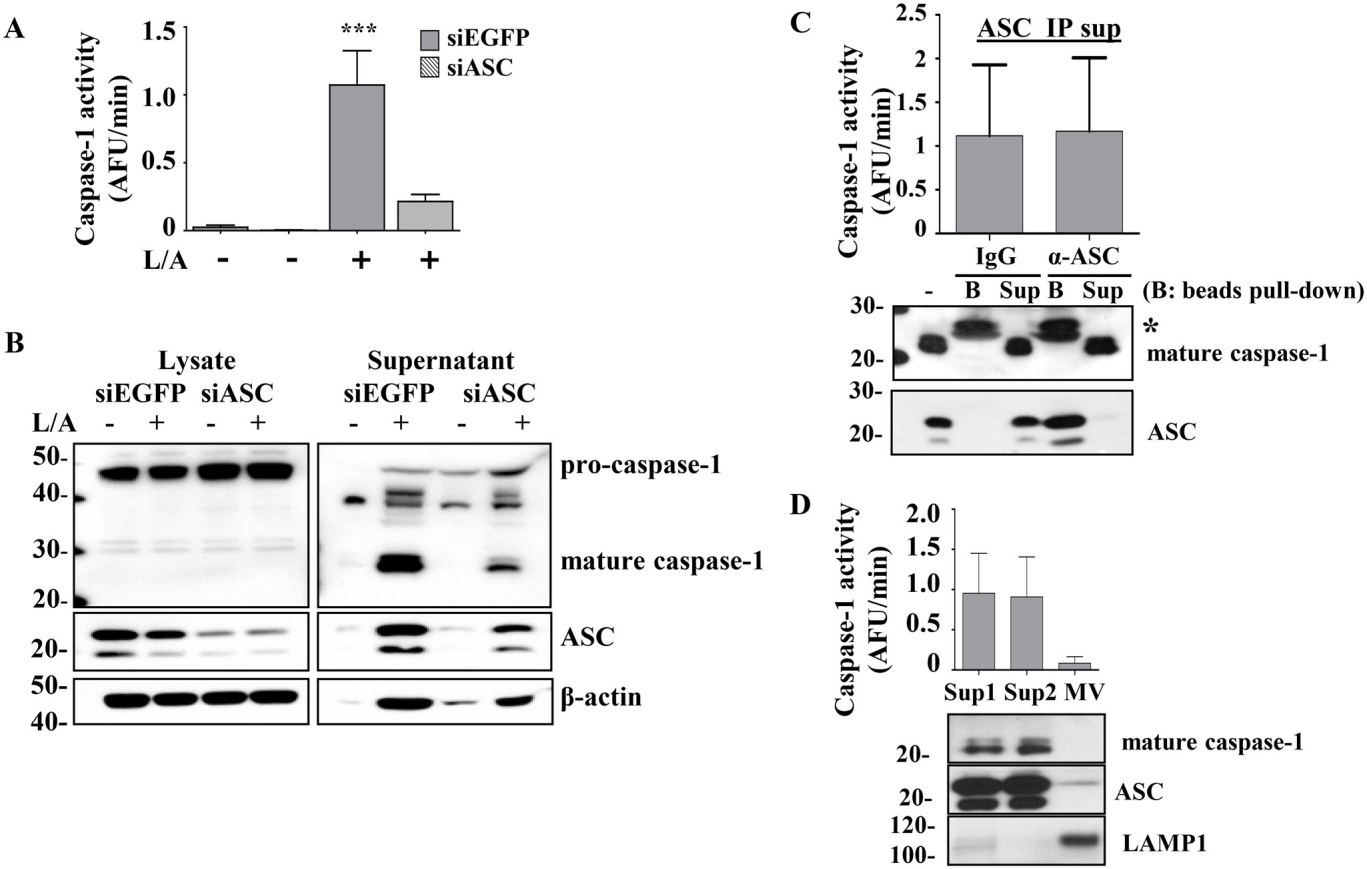
Release of mature caspase-1 is ASC dependent but extracellular caspase-1 activity does not co-precipitate with ASC or predominate in microvesicles. A) THP1 (2x10^7^ cells/ml) cells stably transduced with shRNA for ASC (siASC) and EGFP (siEGFP control) were left untreated or stimulated with LPS/ATP (L/A) for 1h and caspase-1 activity from the supernatants was measured. B) Lysates and supernatants from *(A)* were probed for caspase-1, ASC, and β-actin by immunoblot. C) Supernatants of LPS/ATP treated THP1 cells (2x10^7^ cells/ml) were analyzed for WEHD-afc activity after immunodepletion with either monoclonal anti-ASC antibody (α-ASC) or control mouse IgG1A (IgG) (top panel). Immunoblots for caspase-1 and ASC confirm effective ASC depletion (bottom panel), immunoprecipitate beads **(B)** and residual supernatant **(Sup)**. Co-immunoprecipitation of caspase-1 and immunoprecipitation of ASC are shown below. Original supernatant (-), immunoprecipitate beads (B) and residual supernatant (Sup). * indicates IgG light chain. D) Supernatants of LPS/ATP treated THP1 cells (2x10^7^ cells/ml) were subjected to microvesicle purification by differential centrifugation. Sup1 (control, 16,000g supernatant), Sup2 (100,000g supernatant), MV (100,000g pellet). Caspase-1 activity was measured in the MV-enriched (Sup1), MV-depleted (Sup2), and MV pellet. Fractions were run on immunoblot and LAMP-1 was used as a marker to confirm microvesicle enrichment [37]. Representative gel and data expressed as mean ± SEM from n = 3 independent experiments (A, B, C and D).

Recently, it has been shown that the large ASC specks (>1μm) can be externalized to serve in the propagation of inflammasome activation in a “prion-like” fashion [33–35]. The role of the adaptor protein ASC in forming the NLRP3 inflammasome complex in response to LPS and ATP is well characterized [36]. To confirm the role of ASC in the release of mature caspase-1, we generated stable ASC-deficient THP1 cells (siASC THP1 cells). Knockdown of ASC resulted in decreased processing and release of mature caspase-1 and the detection of lower caspase-1 activity extracellularly as compared to the control siEGFP-stably transduced THP1 cells (Fig 8A and 8B).

In order to elucidate whether the extracellular caspase-1 was bound to ASC complexes, we attempted to co-immunoprecipitate (co-IP) caspase-1 with ASC. Although we were able to immunodeplete ASC from the supernatants, we did not detect mature caspase-1 in the ASC immunoprecipitate (Fig 8C). Due to potential technical limitations of the co-IP approach. we next sought to determine whether the remaining caspase-1 activity in the supernatants decreased following the ASC immunoprecipitation. We found that depletion of ASC from the supernatant had no effect on the caspase-1 activity in the supernatants.

The release of caspase-1 in microvesicles has recently been shown [27]. To identify whether the supernatant caspase-1 activity was coming from microvesicles, we removed microvesicles from the supernatants by differential centrifugation and using LAMP-1 as a marker of microvesicle enrichment as previously described [27, 37]. We found that the supernatant WEHD-afc activity was not diminished by removal of the microvesicle fraction (Fig 8D).

### Immunodepletion does not diminish caspase-1 activity

**Fig 9:**
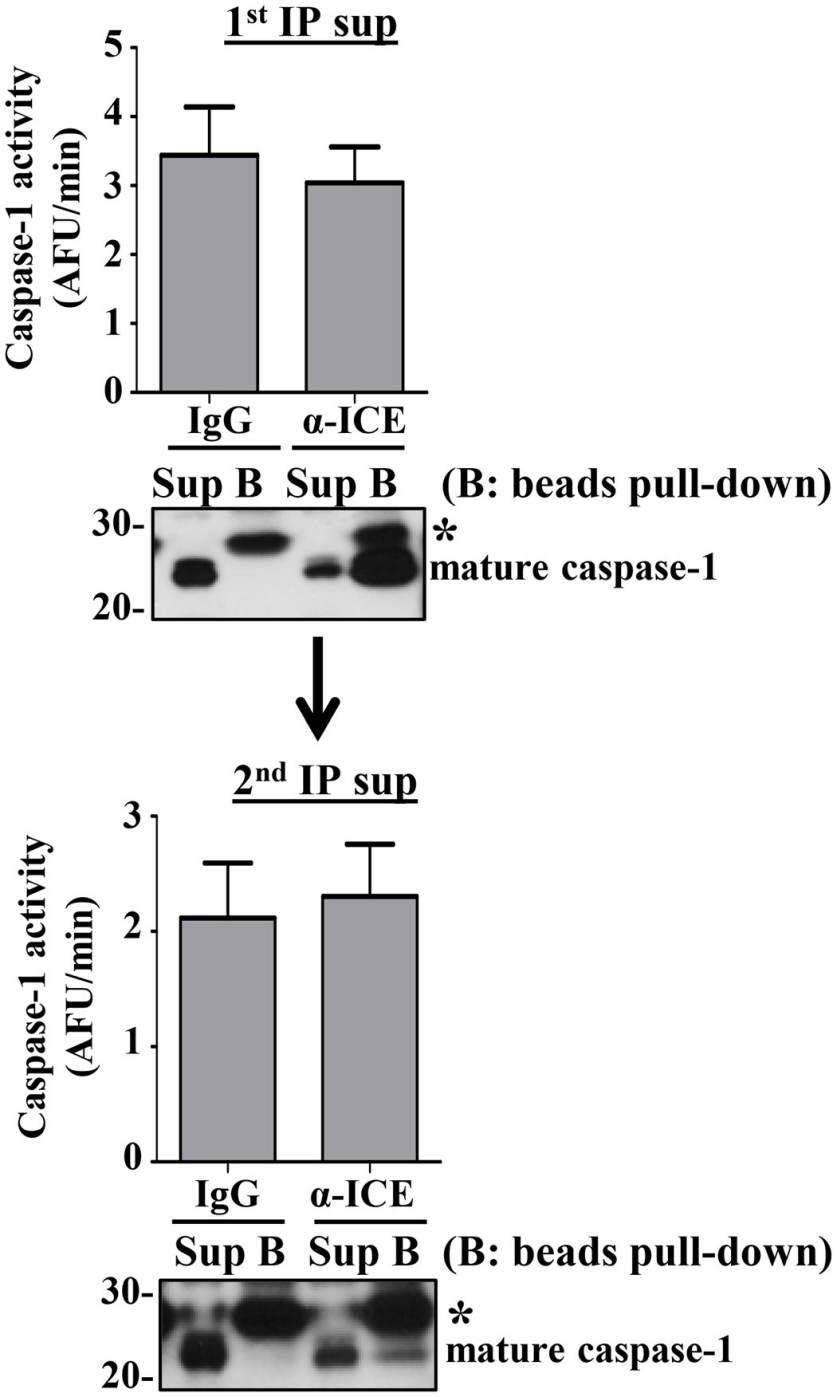
Immunoprecipitation (IP) of caspase-1 does not deplete all caspase-1 or affect detectable activity. Supernatants of LPS/ATP treated THP1 cells (2x10^7^ cells/ml) were analyzed for WEHD-afc activity after two successive caspase-1 immunoprecipitations with either control IgG (IgG) or anti-caspase-1 antibody (α-ICE) (first i.p. top and second i.p. bottom). Immunoblots of caspase-1 from the residual supernatant (Sup) and immunoprecipitate beads (B) are shown for each I.P. * indicates IgG light chain. Data expressed as mean ± SEM from n = 3 independent experiments, representative gels from n = 6 independent experiments.

Next, we attempted to determine whether immunodepletion of caspase-1 from the supernatants would decrease the measured WEHD-afc activity. To our surprise, immunodepletion of caspase-1 from the supernatant did not deplete caspase-1 activity (Fig 9). However, assessing the efficiency of our immunodepletion, we found that we were unable to completely deplete caspase-1 from our samples (Fig 9, ***top blot***). Repeating the immunoprecipitation on these partially depleted sample, we again demonstrated that the remaining caspase-1 did not immunoprecipitate and the activity was undiminished (Fig 9, ***bottom***). The inability to immunoprecipitate the extracellular caspase-1 during the repeat immunoprecipitation was not due to saturation of the antibody, as observed on the first round. We concluded that the active fraction of extracellular caspase-1 might exist in a form that made it inaccessible to immunoprecipitation, in contrast to the caspase-1 that was immunoprecipitated.

### Mature caspase-1 activity exists in a high molecular weight complex

**Fig 10:**
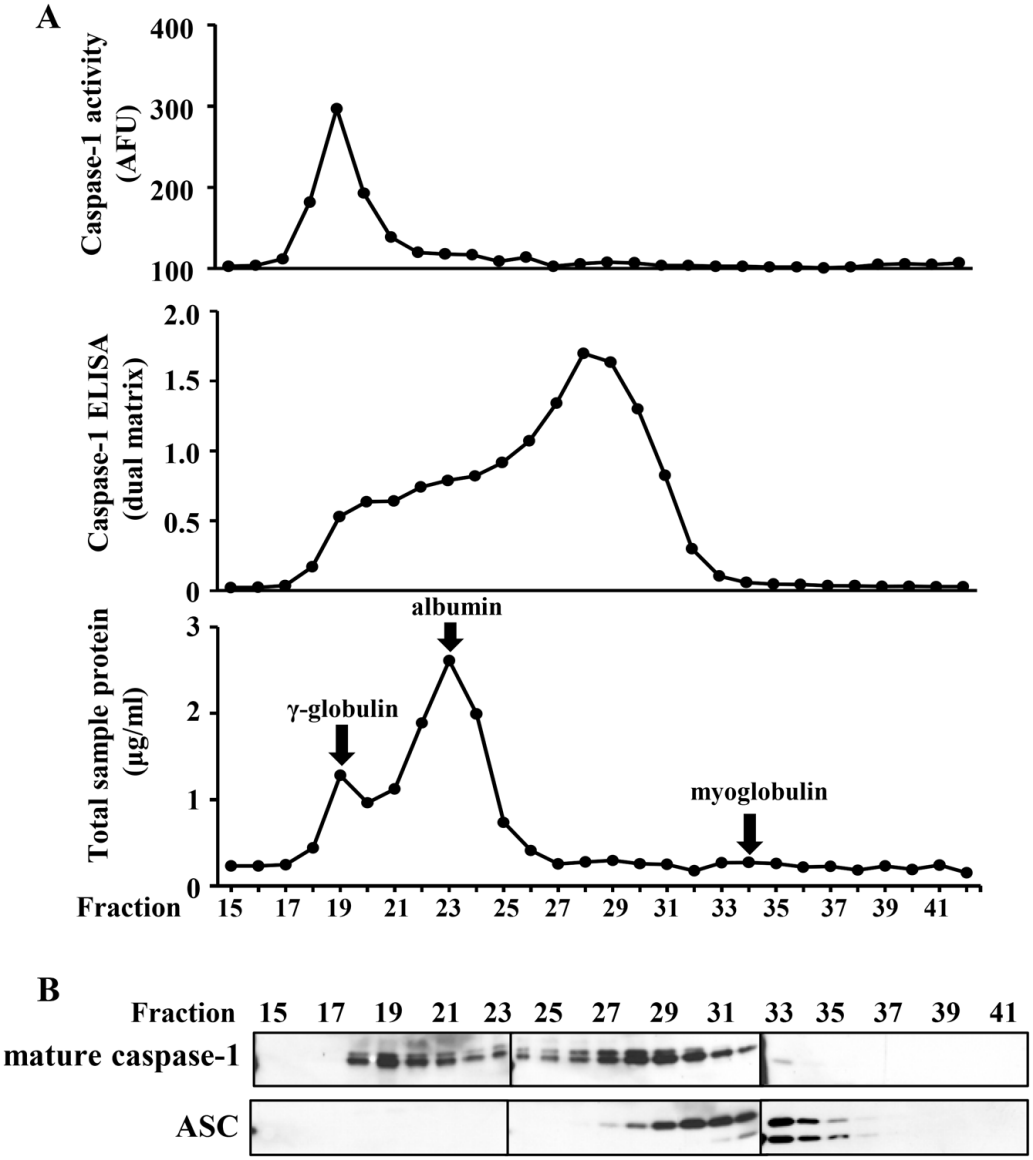
Active caspase-1 is found in a high molecular weight complex. A) Pooled and concentrated supernatant from LPS/ ATP treated THP1 cells (2x10^7^ cells/ml) were fractionated on a HiPrep 16/60 Sephacryl S-200 HR chromatography column. Fractions (2 ml) were collected and analyzed for WEHD-afc activity, detection of caspase-1 by ELISA, and for total protein concentration in the respective fractions. Gamma-globulin (180 kDa), albumin (66 kDa), and myoglobulin (17 kDa) were run on the column as MW markers as indicated. AFU = arbitrary fluorescent units, AU = absorbance units. B) Immunoblots for mature caspase-1 and ASC in the column fractions (15–41) are shown below. Data and representative blots are from n = 2 experiments.

Finally, we subjected the LPS/ATP treated monocyte supernatants to S-200 Sephacryl gel chromatography. Fractions were tested before denaturation for WEHD-afc activity and for antigenic caspase-1 detection by ELISA, and after denaturation by SDS-PAGE generated immunoblots. Of note, p20 caspase-1 was found in two peaks by immunoblots: one in the void volume of the column (≥200kDa) that was associated with WEHD-afc activity and the other in a broad peak that was centered at about 60 kDa (consistent with a p20_2_/p10_2_ dimeric form) (Fig 10). Notably, only the high molecular weight fractions contained the WEHD-afc cleavage activity. In contrast, the lower molecular weight fraction was strongly detected by ELISA. This finding suggests that the stable, released caspase-1 activity exists in a protein complex that may preserve its half-life.

## Discussion

The study of caspase-1 activation has yielded many intriguing insights into a tightly regulated inflammatory process. Here we demonstrate that in response to a strong inflammatory trigger, LPS and ATP, active caspase-1 is released from human monocytes into the extracellular milieu in a functionally stable form. While the release of mature caspase-1 is commonly accepted, to our knowledge the demonstration of stable activity outside the cell has not been previously reported. Furthermore, the stable activity of this released form contrasts to the short half-life of cytosolic caspase-1. Thus, in distinction to apoptotic caspases which are active in the intracellular compartment, active caspase-1 is specifically released into the supernatant but highly regulated in the cytosol [22].

We began this working using the cell-extract model as a means of studying the functional behavior of caspase-1. In the process, we confirmed the observation of Enari et al. that caspase-1 in cytosolic extracts is rapidly activated but loses function compared to the delayed activation and the stability of caspase-3 in the same extracts [28]. The authors proposed that the rapid loss of caspase-1 function may be due to the existence of an endogenous caspase-1 inhibitor, similar to those identified for the apoptotic caspases, such as the inhibitor of apoptosis (IAP) family of proteins [21, 22]. For example, the poxvirus CrmA protein has been shown to interact with and inhibit caspase-1[38]; however, the role of inhibitors in regulating caspase-1 activity is not well understood.

Using the cell-extract technique in THP1 cells [4, 8] we noted that diluting the protein prevented the activation step of caspase-1. However, once activated, caspase-1 induced robust activity that was totally lost within an hour. Initially we considered that the rapid loss of function might be due to degradation of the enzyme into the p20 and p10 subunits and that the highest activity occurred during the transition from zymogen to the mature protein. This concept is however challenged by the stable activity of the mature enzyme detected by the p20 subunits present in the released form of caspase-1.

How caspase-1 becomes endogenously activated in the cell-extract is unknown. There are many parallels between the cell-extract model and what we understand about how the inflammasome becomes activated in live cells. Caspase-1 activation in the cell-extract is facilitated by hypokalemic conditions [39], similar to the central step of NLRP3 inflammasome assembly by K^+^ efflux [40, 41]. It has been proposed that the high concentration of caspase-1 in the cell-extract induces self-dimerization and auto-cleavage [22, 42]. However, in the absence of the adaptor protein ASC this does not occur, confirming that ASC is necessary for this caspase-1 cleavage [6].

Although the cell-extract model is instructive to the function of the inflammasome, we turned to the intact cell model (LPS/ATP activation in THP1 cells) to better characterize the mechanisms of caspase-1 regulation. As expected, caspase-1 was released in parallel with ASC and the processed form of IL-18. However, we were surprised to find that mature caspase-1 released outside the monocyte exists in two forms, an active, non-immunodepleteable form, and an inactive, immunoprecipitatable form. The released caspase-1 is detectable as the p20 processed form by immunoblot and contains the ability to cleave the sensitive caspase-1 substrate WEHD-afc and to convert 31kDa proIL-1β to the 28 kDa cleaved form which is step one in IL-1β processing [32]. However, confirmation that this WEHD-afc activity is due to caspase-1 could not be confirmed by immunodepletion studies. Although we could remove a significant amount of caspase-1, we found that we could not remove the WEHD-afc activity.

We hypothesized that the released caspase-1 that had WEHD-afc activity might be bound in a complex that prevented its detection by antibody. To address this concern we subjected the supernatant to size exclusion chromatography. As we hypothesized, the WEHD-afc activity eluted along with p20 caspase-1 at the void volume suggesting a protein complex (≥ 200 kDa) that has sequestered active caspase-1. In contrast the lower molecular weight peak of p20 caspase-1 did not retain WEHD-afc activity. Thus, extracellular caspase-1 may be present in a complex that stabilizes its function. Nevertheless, we could label a p20 band in the supernatant with biotin-YVAD-cmk, an irreversible inhibitor that binds to the active site, suggesting that the activity was indeed due to caspase-1. However, it is possible that another 20 kDa protease was also labeled by YVAD-biotin.

There are limitations to our current findings that need to be addressed. First of all, it is conceivable that the p20 caspase-1 in the high molecular weight complex is not the protease responsible for the WEHD-afc activity. Another noncaspase-1 protease may co-elute in the fraction and be responsible for the WEHD-afc activity. However, the ability of YVAD-cmk to inhibit the activity and the YVAD-biotin labeling of a p20 band strongly supports a role for caspase-1. Furthermore, we have yet to characterize the proteins present in the high molecular weight complex with caspase-1. Future studies will be directed at understanding the nature of this high molecular weight WEHD-afc cleaving activity.

We do know that caspase-1 activation and the release of mature IL-1β and IL-18 in response to inflammasome stimuli are tightly linked. It is not known how mature IL-1β and IL-18 are released from the monocyte as they lack the secretory peptide sequence which would target them for classical secretory pathways [36, 43]. Similarly, mature caspase-1 and ASC are released simultaneously but we were unable to co-immunoprecipitate them in the supernatant [20]. The absence of the CARD pro-domain on mature caspase-1 prevents its association with ASC. It is conceivable that caspase-1 cleavage of proIL-1β/IL-18 requires a yet to be described complex that puts the active released caspase-1 into a favorable relationship with these precursor cytokines.

The ability of extracellular caspase-1 to generate the first cleavage step in pro-IL-1β processing suggests that cytokine and enzyme interaction requires facilitation by compartmentalization that links processing to the release process [44]. The finding that caspase-1 remains functional extracellularly but is inaccessible to antibodies implies a steric hindrance. This type of steric hinderance by a large complex has been observed previously with neutrophil elastase [45]. Neutrophil elastase can be captured by two distinct protease inhibitors, alpha-2 macroglobulin and alpha-1 antitrypsin. When interacting with the broad spectrum protease inhibitor, alpha-2 macroglobulin, it remains accessible to small substrates but not to macromolecules like elastin and is not detectable by ELISA [45]. In contrast, when bound to alpha-1 antitrypsin, neutrophil elastase is nonfunctional but accessible to immunodetection. A similar phenomenon could explain the presence of a functional but non-immunoglobulin accessible form of caspase-1, together with the nonfunctional, immunoglobulin accessible form.

Further investigation is required to understand the implications of releasing mature caspase-1 into the extracellular environment, as well as to characterize how it is inhibited in cytosolic environments. These findings serve as a foundation for subsequent studies designed to explore these processes in a more physiological setting that have the potential to yield additional insights into the functions of caspase-1.
